# Untangling an AGS Outbreak Caused by the Recombinant GII.12[P16] Norovirus With Nanopore Sequencing

**DOI:** 10.3389/fcimb.2022.911563

**Published:** 2022-07-05

**Authors:** Qianling Xiong, Huimin Jiang, Zhe Liu, Jinju Peng, Jing Sun, Ling Fang, Caixia Li, Ming Qiu, Xin Zhang, Jing Lu

**Affiliations:** ^1^School of Public Health, Southern Medical University, Guangzhou, China; ^2^Guangdong Provincial Institution of Public Health, Guangdong Provincial Center for Disease Control and Prevention, Guangzhou, China; ^3^Guangdong Provincial Center for Disease Control and Prevention, Guangzhou, China; ^4^Haizhu Provincial Center for Disease Control and Prevention, Guangzhou, China; ^5^School of Public Health, Sun Yat-Sen University, Guangzhou, China

**Keywords:** norovirus, nanopore, metatranscriptomic sequencing, GII.12[P16], phylogenetic, recombination

## Abstract

For a rapidly spreading virus such as NoV (norovirus), pathogen identification, genotype classification, and transmission tracing are urgent for epidemic control. Here, we applied the Nanopore metatranscriptomic sequencing to determine the causative pathogen of a community AGS (Acute gastroenteritis) outbreak. The results were also confirmed by RT-PCR. The NGS (Next Generation Sequencing) library was constructed within 8 hours and sequence analyses were carried out in real-time. NoV positive reads were detected in 13 of 17 collected samples, including two water samples from sewage treatment tank and cistern. A nearly complete viral genome and other genome fragments could be generated from metatranscriptomic sequencing of 13 samples. The NoV sequences from water samples and cases are identical suggesting the potential source of the outbreak. The sequencing results also indicated the outbreak was likely caused by an emerging recombinant GII.12[P16] virus, which was only identified in the United States and Canada in 2017–2018. This is the first report of this emerging variant in mainland China, following the large outbreaks caused by the recombinant GII.17[P17] and GII.2[P16] in 2014 and 2016, respectively. Closely monitoring of the prevalence of this recombinant strain is required. Our data also highlighted the importance of real-time sequencing in emerging pathogens’ surveillance.

## Introduction

The real-time surveillance of infectious viruses in an outbreak is critical for epidemic control. Traditional diagnosis relies on techniques including the isolation of microorganisms in culture, detection of pathogen-specific antibodies (serology) or antigens, and molecular identification of microbial nucleic acids (DNA or RNA) (Pankaj, 2021). The major limitations are the long-term duration (the culture method) and the requirement of prior knowledge on detecting pathogens (the specific primers for PCR or the specific antibodies for serological methods). Therefore, a limited number of pathogens are conventionally detected. Next-generation sequencing (NGS) has been demonstrated for broad-spectrum pathogen detection and genomic surveillance of viral outbreaks. Compared to traditional methods, NGS approaches characterize all DNA or RNA present in a sample enabling analysis of the entire microbiome and the human host genome or transcriptome in patient samples. However, disease outbreaks or epidemics always progress rapidly and the application of NGS is limited due to the long duration time in library construction, sequencing, and data analysis. Nanopore sequencing is a third-generation sequencing method that enables rapid NGS library construction and a real-time sequencing analysis (Greninger et al., 2015; Kafetzopoulou et al., 2018; Kafetzopoulou et al., 2019; Lewandowski et al., 2019; Chan et al., 2020; Schuele et al., 2020).

Norovirus is highly contagious and has a great genetic diversity. According to the nomenclature system of NCWG (Norovirus Classification Working Group), NoV can be classified into ten genogroups (GI–GX) and two tentative genogroups based on the VP1 coding sequences. These genogroups could be further subdivided into 49 confirmed capsid genotypes based on the complete VP1 gene sequences and 60 confirmed P-types based on the nucleotide sequences of RdRp gene (Chhabra et al., 2019). GII.4 has been identified as the most predominant genotype worldwide from 1995 to 2013, and novel GII.4 variant has emerged every 2-5 years leading to global epidemics (Tohma et al., n.d.; Vega et al., 2014). In 2014 and 2016, the novel genetic variants GII.17[P17] and the recombinant GII.2[P16] were identified in Guangdong, China (Lu et al., 2015, n.d.). The dominance of these new variants has resulted in increasing AGS outbreaks in local (Lu et al., 2015, n.d.) and the global transmission of these variants were also identified (van Beek et al., 2018). Therefore, the genetic surveillance of NoV diversity in the high-risk region should be warranted for the early warning of global epidemics.

In June 2020, an AGS outbreak occurred in a community in Guangdong, China, with up to 217 cases. We used the metatranscriptomic with Nanopore sequencing to identify NoV as the causative agent of this AGS outbreak. The sequencing method provided similar sensitivity to the specific RT-PCR. Further, the real-time generated sequences rapidly inferred the potential source of this outbreak. The assembled viral sequence indicated the virus as a recombinant NoV variant which may be imported into mainland China recently.

## Materials and Methods

### Sample Collections

The outbreak-related cases were defined as ≥3 episodes of diarrhea and ≥2 episodes of vomiting in the 24 hours in the community from May 10 to June 13. According to the Guangdong provincial AGS outbreak surveillance scheme (Lu et al., 2015; Lu et al., 2017), an outbreak with a cluster of at least 10 AGS cases (meeting the Kaplan criterion) within 3 days must be reported to Guangdong Provincial Center for Disease Control and Prevention (GDCDC). Epidemiology information was collected for the outbreak-associated cases. Anal swabs from 15 symptomatic cases and water samples from the sewage treatment tank and the cistern were collected for laboratory testing and tracing of the potential source of the outbreak. (**Figure 2A**, created with BioRender.com).

### Pretreatment of Samples, Nucleic Acid Extraction, RT-PCR and Viral Load Calculation

Conventional pre-swabbing methods performed anal swabs. Water samples were concentrated for viruses by negative-charged filter membrane absorption and ultrasonic methods (Lu et al., 2021). 140μL of each of the obtained samples were used to extract total viral RNA using the QIAamp Viral RNA mini kit (QIAGEN Co., Ltd.) according to the instructions. Subsequently, 5μL of total RNA was used for Quantitative Real-time PCR (RT-PCR) diagnosis of NoV nucleic acid (MABSKY Co., Ltd.). Viral load calculation was performed as previously described (Li et al., 2022). NoV positive RNA with the absolute viral copies determined by droplet digital PCR (ddPCR) was ten-fold serially diluted (from 1.43×10^5^ to 1.43 copies/ul) and undertook RT-PCR in the same condition of the other clinical samples. A standard curve was built with the viral copies and the corresponding Ct values (**Figure S1**). The relative viral copies in each sample were converted to RNA copies per ul according to the Ct value.

### Nanopore Metatranscriptomic Sequencing

SISPA protocol was followed for metatranscriptomic sequencing. Ten μL of total RNA was reverse transcribed with the tagged random primer (Primer A - 5’-GTT TCC CAC TGG AGG ATA-(N9)-3’) by using SuperScript™ IV First-Strand Synthesis System (Thermo Fisher Co., Ltd.) (Lewandowski et al., 2019; Gage and David, 2020). The second-strand synthesis was performed with 1μL of 10X NEB buffer (NEB, Inc.), 0.3μL of 10mM dNTP mix, 6.7μL of nuclease-free water, 2μL of Klenow (NEB, Inc.) and 20μL of single-stranded cDNA for each 30μL of the reaction mixture. Reactions were incubated at 37°C for 30mins, 94°C for 2mins, and then cooled to 4°C. Five μL double strand cDNA was further amplified with 1μL primer B (5′-GTTTCCCACTGGAGGATA-3′) by using Q5^®^ High-Fidelity 2X Master Mix (NEB, Inc.). The amplification steps were 98°C for 30s, followed by 35 cycles of 98°C for 15s, 50°C for 45s, and 72°C for 1min, and complete at 72°C for 5mins. The completed amplification products were purified using AMPure XP beads (Beckman Coulter, Inc.) in a 1:1 ratio and quantified by the Qubit High Sensitivity Double-Stranded DNA kit (Thermo Fisher Co., Ltd.).

Library prepared by modifying the Ligation Sequencing Kit opening protocol (SQK-LSK109, Nanopore Technologies Co., Ltd.) from the Nanopore community (https://community.nanoporetech.com/protocols). In modified protocol, all sequencing samples were mixed after the barcode ligation and then purified in single tube, rather than was purified separately in the standard protocol. In brief, input 0.2pmol of DNA for each sample separately and select a unique barcode from the EXP-NBD104 and EXP-NBD114 native barcode kits. Then, the library was further purified by adding motor proteins to magnetic beads and loaded 75μL sequencing mix (65ng 12μL library, 25.5μL loading beads, 37.5μL sequencing buffer) sequenced for 48 hours on FLO-MIN106 flow cells on the GridION device (Nanopore Technologies Co., Ltd.).

**Figure 1:**
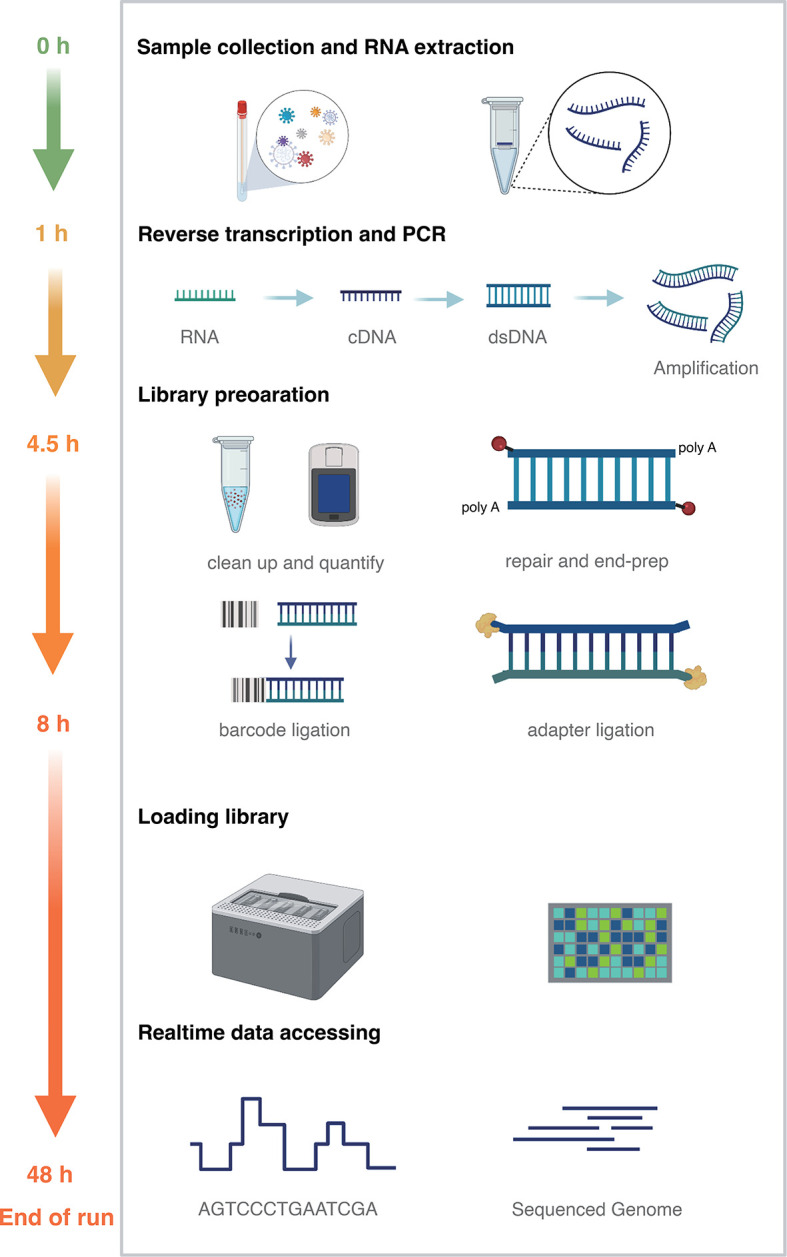
Nanopore metatranscriptomic sequencing method time frame diagram. Sample collection and RNA extraction (green, 1 hour); reverse transcription, second-strand synthesis and PCR (yellow, 3.5 hours); library construction (orange, 3.5 hours); library loading, sequencing, real-time data analysis to end of sequencing (till 48 hours).

Real-time sequence analyses were performed by using guppy (version 5.0) for basecalling (convert fast5 to fastq) and barcode trimming. Single end and paired-ends barcode trimming were tested. The following analysis was carried out on IPH-Nano (version 1.0) (Guangdong Biostar Gene Company). In brief, reads were taxonomically classified by kraken2 (version 2.08-beta) and categorized against the GenBank database (Wood et al., 2019). The reports from kraken2 were used for visualization by Pavian (Breitwieser and Salzberg, 2020) and NoV sequences were extracted from raw sequences by Taxonkit (Version 0.7.2) (Shen and Xiong, 2019). Genotyping was performed by the Norovirus Typing Tool Version 2.0 (Kroneman et al., 2011). Reference sequences were selected from public databases according to genotype (GenBank accession number MK762627). Minimap2 (version 2.17) (Li, 2018) was used to map the merged fastq sequences to the reference genome. Medaka (version 1.0.3) (https://github.com/nanoporetech/medaka) was used for error correction followed by the Longshot (version 0.4.1) for further correction. Python scripts to record sites with sequencing depth <=15 and BCFtools (version 1.10.2) created the consistency sequences (Li, 2011) (**Figure 1**, created with BioRender.com).

### Illumina Metatranscriptomic Sequencing

Metatranscriptomic sequencing for RNA viruses was performed as previously described (Lu et al., 2020). Briefly, RNA was first purified using TURBO DNase (Thermo Fisher Co., Ltd.). Reverse transcription of RNA to cDNA was carried out. cDNA ribosome removal was performed by ZapRv2 (mammalian-specific). The cDNA was then converted to Second-stranded DNA and library preparation was carried out using the SMARTer Stranded Total RNA-Seq Kit v2 (Takara Bio, Inc.), which includes end-repair, A-tailing, and adapter ligation. Sequencing of metatranscriptome libraries was conducted on the Illumina NovaSeq 6000 platform (PE 150) and more than 40,000,000 reads were generated. For Illumina sequencing data, BWA-MEM (version 0.7.17) was used for sequence mapping (Li, 2013). The reference sequence was the same as the Nanopore sequencing analysis. SAMtools (version 1.10) was used to calculate the percentage of reads mapped and the depth of coverage (Li et al., 2009). iVar (version 1.2) called consensus from the aligned BAM file (Grubaugh et al., 2019).

### Phylogenetic Analyses

The whole-genome phylogenetic tree was built with the sequence acquired in this outbreak and 30 sequences closely related by NCBI Blast (https://blast.ncbi.nlm.nih.gov/Blast.cgi). The phylogenetic trees of VP1 and RdRp genes were constructed by including the related VP1 gene sequences of GII.12 and related RdRp gene sequences of GII.P16 (**Tables S2, S3**). Maximum-likelihood (ML) trees were estimated for three datasets separately in IQ-Tree 1.6.12 (Nguyen et al., 2015). Phylogenetic trees were annotated and visualized with ggtree (Yu et al., 2017).

## Results

### Epidemiological Investigation

**Figure 2:**
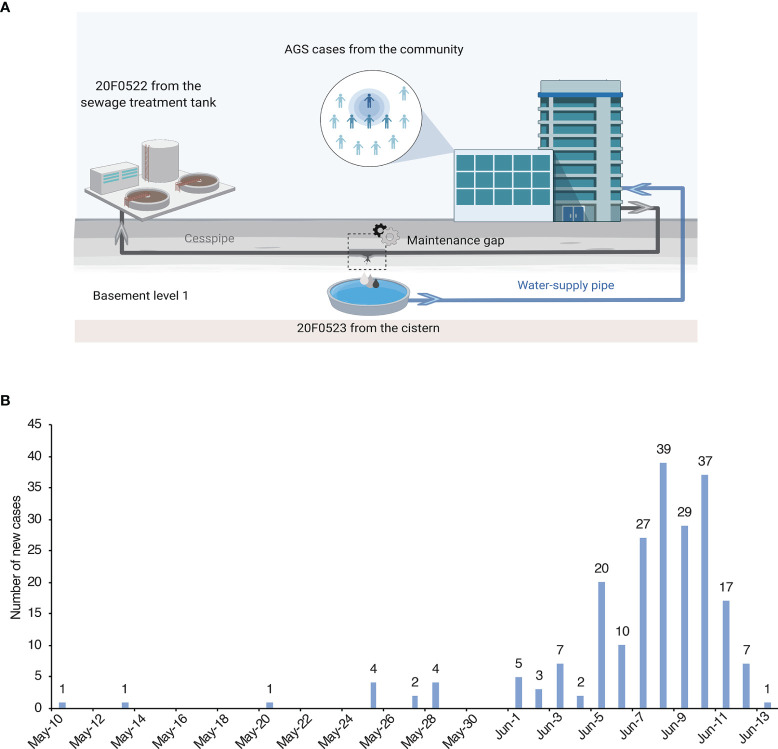
Samples collection locations and daily number of new cases. **(A)** The location of cases and water samples. The cesspipe is parallel over the water supply pipe, and there is a maintenance gap near the reservoir in the basement level 1. **(B)** Daily number of new cases of the epidemic in Guangdong, China during May 10 to June 13, 2020.

The index case developed diarrhoea on May 10^th^ and began vomiting on May 25^th^, 2020. From Jun 5^th^ to June 11^th^, the number of cases was increased sharply from 30 to 209, with the highest number of 39 cases reported on Jun 8. According to Guangdong provincial AGS outbreak surveillance network, a total of 217 associated cases were identified in this outbreak (**Figure 2B**). The reported cases consisted of 51.6% (112/217) females and 48.4% (105/217) males. The age of cases ranged from 5 months to 84 years (median = 31), and 77 cases were pupils and pre-schoolers (30.88%). The most frequently reported clinical symptoms were diarrhoea (184/217, 84.79%), abdominal pain (165/217, 76.04%), and vomiting (90/217, 41.47%). In some cases, fever symptoms (34/217, 15.67%) were observed, with a maximum of 38.5°C (**Table S1**).

### Etiological Investigation

To characterize the causative pathogen, Nanopore metatranscriptomic sequencing and NoV specific RT-PCR had performed simultaneously. A total of 15 anal swabs and two supply water samples were collected on Jun 10^th^ and Jun 13^th^, 2020, of which 12 of 15 swabs and both water samples were tested positive for GII NoV by RT-PCR with Ct values ranging from 30.7 to 36.27 (**Table 1**). Nanopore metatranscriptomic sequencing of all samples (15 swabs and 2 water samples) showed that NoV specific reads were identified in 13 of 17 except 3 RT-PCR negative samples and one anal swab with a relatively high Ct value (36.27, 16 copies/μL). All RNA with viral copies over 26 copies/μL have NoV specific reads ranging from 318-1061 bp been detected by nanopore metatranscriptomic sequencing.

**Table 1:** Ct values, Nanopore sequencing Nov reads extracted and genotype classified results for outbreak, May 10-June 13, 2020, Guangdong, China.

Samples	Ct Value	Relative virus RNA copies/μL	Number of Nov reads extracted		Nov readsaveragelength	Genotype
			Single endtrim barcode	Both endstrim barcode		
20F0507	32.99	137	13	8	535	–
20F0508	31.00	512	576	385	744	GII.12[P16]
20F0509	Negative	–	0	0	0	–
20F0510	34.33	56	48	33	1061	GII.P16
20F0511	32.43	198	250	159	604	GII.12[P16]
20F0512	31.10	479	1045	758	826	GII.12[P16]
20F0513	35.27	30	4	3	318	–
20F0514	32.33	212	29	19	622	GII.P16
20F0515	31.81	299	5	3	832	–
20F0516	Negative	–	0	0	0	–
20F0517	36.27	16	0	0	0	–
20F0518	35.47	26	74	49	623	–
20F0519	31.49	370	37	24	738	GII.12[P16]
20F0520	Negative	–	0	0	0	–
20F0521	30.70	624	234	188	673	GII.12[P16]
20F0522*	34.53	49	4	4	1134	–
20F0523*	33.44	102	3	2	470	GII.P16
NC	Negative	–	3	0	0	–

NC: Negative control (Nuclease-free water).

*: Water samples.

-: Cannot classified.

Notably, paired-ends barcode trimming should be compulsory for the sequencing data analysis. As shown in **Table 1**, the false positive may be caused by barcode bleeding when only single end barcode trimming was applied. Five samples, having NoV specific reads (average length of ~ 710 bp) mapped to RdRp and VP1 regions, could be classified as GII.12[P16] (**Table 1**). The nearly complete viral genome (coverage 80.3%) could be assembled from the sequencing result of 20F0512 which included 758 NoV specific reads. Two clinical cases and one water sample comprising sequencing reads mapped to the RdRp and could be classified as GII.P16. Without reads mapped to VP1 region, the VP1 genotype of NoV in these three samples could not be defined. The NoV related reads from all these samples shared 100% sequence identity with 20F0512 suggesting the outbreak was highly likely caused by a common source.

To prove the sequence accuracy generated from Nanopore sequencing, we also performed the metatranscriptomic sequencing of the case 20F0512 on the Illumina platform. A total of 45,617,813 reads were generated in the Illumina platform, of which 90.7% reads had a minimum base quality score Q30. A total of 3754 reads (average length ~ 118 bp) could be mapped to the reference genome, with sequencing coverage at 79.2%. There is no sequence variance observed between the consensus sequence generated from the Nanopore and Illumina platform, suggesting that the pipeline used in data processing and relative high sequencing depth could correct the potential random sequencing errors during nanopore sequencing.

### Phylogenetic Analyses

**Figure 3:**
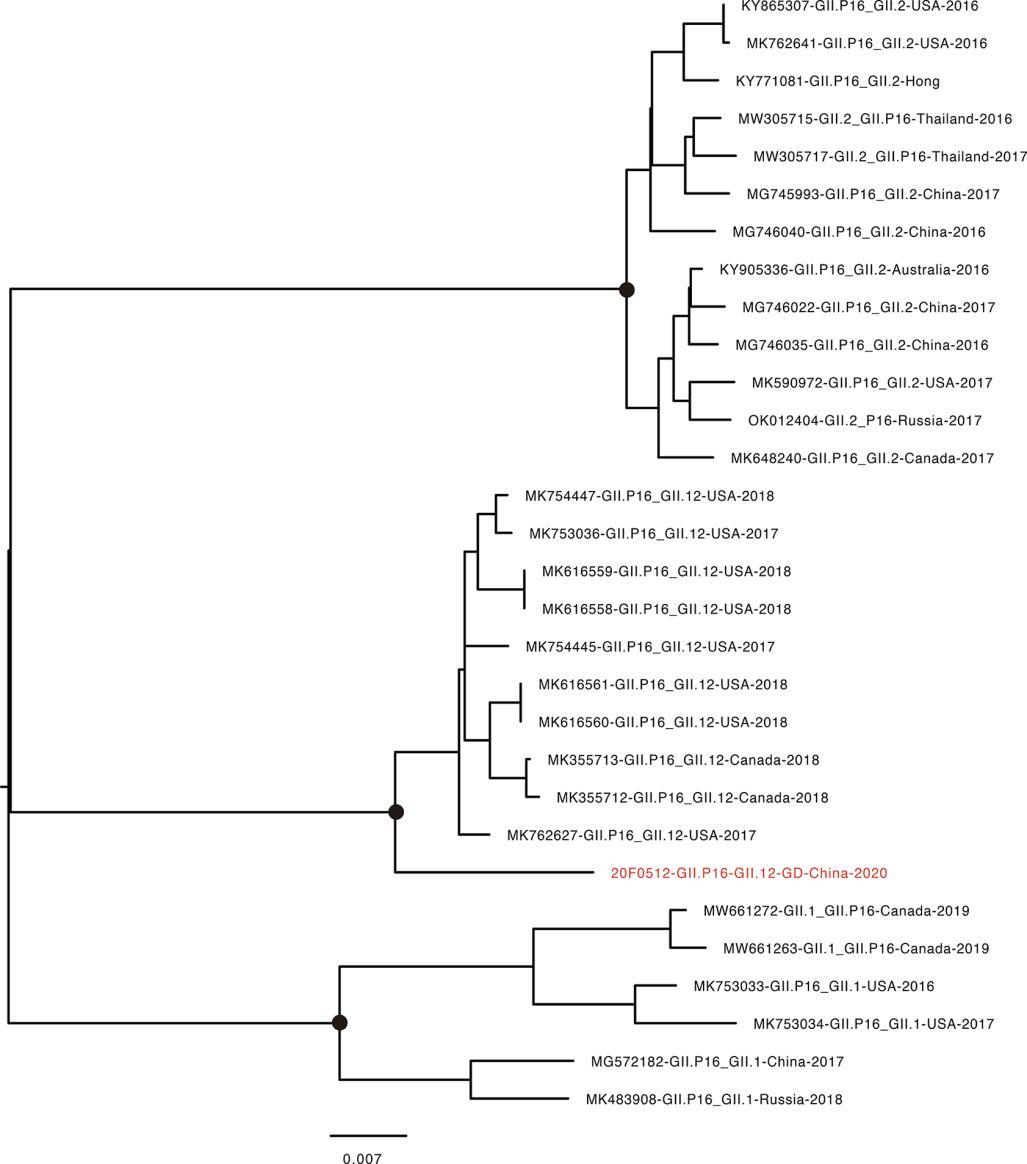
Phylogenetic tree of whole-genome for NoV GII.12[P16]. The sequence highlighted in red indicates the GII.12[P16] strain found in the current epidemic. GenBank accession numbers, genotype, the country identified, and year identified are provided. Scale bar represents the number of differences between sequences. Black dots indicate bootstrap support >80 at the root node of selected clade.

**Figure 4:**
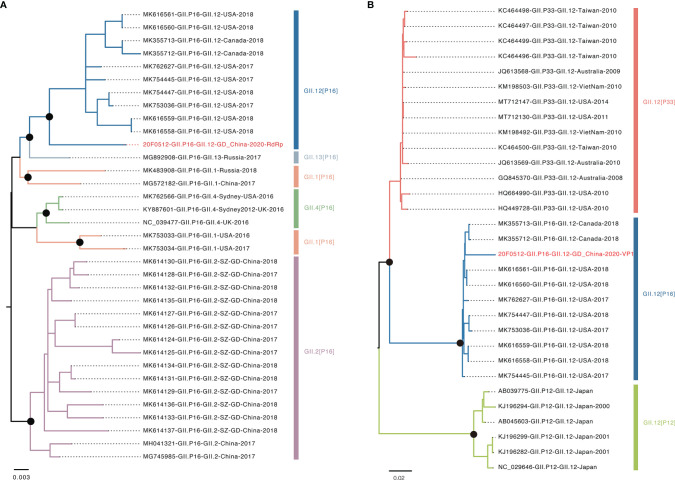
Phylogenetic analysis of VP1 and RdRp genes. **(A)** Phylogenetic tree of RdRp region. Different colors indicated various genotype. The red highlighted sequence represents RdRp gene of this outbreak. **(B)** Phylogenetic tree of VP1 region. The red highlighted represents VP1 gene of this outbreak. Black dots indicate bootstrap support >80 at the root node of selected clade.

The recombinant GII.12[P16] was first identified in the United States in 2017 (Barclay et al., 2019). The endemic circulation of this recombinant variant was reported in the United States and Canada 2017-2018 (Pabbaraju et al., 2019). The whole-genome sequence analysis showed the GII.12[P16] identified in Guangdong 2021 was clustered with the previous strains identified in the United States and Canada, sharing 96.99% sequence similarity to the closely related strain (MK762627, **Figure 3**). The phylogenetic trees of VP1 and RdRp were constructed with the closely related sequences (**Figure 4**, **Tables S2, S3**). For VP1 gene, there were a few of GII.12 VP1 gene sequences in public database suggesting the limited circulation or surveillance of this genotype. None of GII.12 VP1 sequences were collected from mainland China. Consistent with the whole genome sequence analysis, the VP1 phylogeny indicated the Guangdong GII.12 outbreak strain was closely related with strains reported in the United States and Canada 2017-2018. For RdRp gene, the Guangdong outbreak strain was also clustered with the GII.12[P16] strains reported in the United States and Canada, and separated from the local dominant circulating genotype GII.2[P16]. Notably, the frequent recombination could be inferred as GII.1[P16], GII.2[P16], GII.4[P16], GII.12[P16] and GII.13[P16] strains were separately clustered in GII.P16 RdRp phylogeny and GII.12[P12], GII.12[P16] and GII.12[P33] strains were separately clustered in GII.12 VP1 phylogeny (**Figure 4**, **Table S3**).

## Discussion

Norovirus is highly contagious and has a great genetic diversity. Methods for rapid NoV genotyping, which are critical for epidemic control and early warning, are still very limited. In this study, we determined and characterized the causative agent of an AGS outbreak by using metatranscriptomic sequencing on the Nanopore platform. The Nanopore metatranscriptomic sequencing showed similar sensitivity with the RT-PCR in detection of NoV in both clinical samples and water samples (**Table 1**). More important, the real-time sequencing inferred that the outbreak was of single origin and the emerging recombinant NoV variant was the potential causative pathogen. Our study result provides a solution for timely pathogen surveillance, sequence and transmission chain analysis during AGS outbreaks, which won valuable time for outbreak control.

For metatranscriptomic sequencing, Illumina has been the most widely used platform with high accuracy (~0.1% base calling error) (Goodwin et al., 2016). Its short length (75-300 bp), when dealing with metatranscriptomic sequences data, usually leads to highly fragmented genomes, which complicates the analysis (Latorre-Pérez et al., 2020). And its sequencing period is too long which takes more than 20 hours and data must be obtained after completing the sequencing (Greninger et al., 2015). This cannot be satisfied for outbreaks that require rapid identification of pathogens. Nanopore metatranscriptomic sequencing allows library preparation to be completed within 8 hours, and the sequencing process can be analyzed in real-time. The presence of the particular pathogen in multiple samples could be immediately identified, making it easier to determine the outbreak’s possible cause and provide timely guidance for epidemic management. Despite the high errors inherent in Nanopore sequencing platforms, the recently developed basecalling model and a relatively high reading depth can overcome these limitations (Zeng et al., 2020). Meanwhile, the advantage of Nanopore sequencing, the relative long length of reads (average specific NoV reads more than 710bp), is beneficial to the sequence assembly and the recombination analysis. Our study result demonstrated there is no sequence variance between the consensus sequence generated from the Nanopore and Illumina platform for positions with a relatively high sequencing depth (>15).

This is the first identification of the recombinant GII.12[P16] in mainland China following the large outbreaks caused by the recombinant GII.17[P17] and GII.2[P16] in 2014 and 2016 (Lu et al., 2015, n.d.), respectively. This variant was suggested to be endemically circulating in the United States in 2017 and Canada in 2018, but has not been identified in other regions (Barclay et al., 2019; Pabbaraju et al., 2019). Our results highlighted the epidemic risk of this emerging variants, and the divergence between the Guangdong strains and the relative strains identified in the U.S. and Canada indicating the hidden circulation of this variant in the population. In addition, the frequent recombination was noted from the phylogeny of RdRp and VP1 genes. Particularly, the P16 RdRp sequences have been adopted by multiple NoV VP1 genotypes including the dominant circulating genotypes GII.4 and GII.2. Regarding the large outbreaks caused by the recombinant variant of GII.2[P16] in Guangdong, China, continuous genetic surveillance and close monitoring of the prevalence of this emerging variant GII.12[P16] is warranted.

## Data Availability Statement

Sequencing data were deposited in the GSA database of the National Genomics Data Center (https://bigd.big.ac.cn/) with submission number CRA006447 (https://ngdc.cncb.ac.cn/gsa/browse/CRA006447).

## Ethics Statement

The studies involving human participants were reviewed and approved by the Institute ethics committee, Guangdong Provincial Center for Disease Control and Prevention, Guangzhou. Written informed consent for participation was not required for this study in accordance with the national legislation and the institutional requirements.

## Author Contributions

JL, QX, and XZ designed the study. QX wrote the manuscript and performed genetic analyses. QX, HJ, JS, LF, MQ and CL undertook the experiments. JP, JL and ZL edited the manuscript. All authors listed have made a substantial, direct, and intellectual contribution to the work and approved the submitted version.

## Funding

This study was supported by grants from The Key Research, Development Program of Guangdong Province (2019B111103001), Guangdong Medical Research Program (A2020052) and Guangzhou Science and Technology Project (202102080590).

## Conflict of Interest

The authors declare that the research was conducted in the absence of any commercial or financial relationships that could be construed as a potential conflict of interest.

## Publisher’s Note

All claims expressed in this article are solely those of the authors and do not necessarily represent those of their affiliated organizations, or those of the publisher, the editors and the reviewers. Any product that may be evaluated in this article, or claim that may be made by its manufacturer, is not guaranteed or endorsed by the publisher.
