# Supplementary material for: Untangling an AGS Outbreak Caused by the Recombinant GII.12[P16] Norovirus With Nanopore Sequencing
Source: Front Cell Infect Microbiol. 2022 Jul 5;12:911563. doi: 10.3389/fcimb.2022.911563 (PMC9294139; doi:10.3389/fcimb.2022.911563)
Supplement: Supplementary file 1 [file DataSheet_1.docx]

Supplementary materials

## Supplementary Figures

**Supplemental Figure 1.** Standard curve relationship between virus RNA copies and Ct values. Absolute quantification of norovirus by ddPCR was ten-fold serially diluted (from 1.43×10^5^ to 1.43 copies/μl). The viral RNA was evaluated with RT-qPCR. The best-fit linear regression of the average Ct value on the log_10_-transformed standard values had slope -0.2879 and intercept 11.634 (R^2^ = 0.9985).

## Supplementary Tables

Table S1. Demographic and clinical characteristics of the associated cases in this AGS outbreak

| Demographics | n=217 |
| --- | --- |
| Age (median, range) | 31 (0.4~84) |
| Gender |  |
| Male | 105(48.4%) |
| Female | 112(51.6%) |
| Symptoms |  |
| Diarrhoea | 184 (84.79%) |
| Abdominal pain | 165 (76.04%) |
| Vomiting | 90 (41.47%) |
| Fever | 34 (15.67%) |
| Test and outcome |  |
| Self-medication | 148(68.20%) |
| Outpatients | 42(19.35%) |
| Untreated | 27(12.44%) |
| Hospitalization and death | None |

Table S2. GenBank accession numbers and norovirus dual-typing information downloaded from Genbank for VP1 phylogenetic tree construction

| Genbank accession No. | VP1 | RdRp | Genbank accession No. | VP1 | RdRp |
| --- | --- | --- | --- | --- | --- |
| GQ845370 | GII.12 | GII.P33 | HQ664990 | GII.12 | GII.P33 |
| JQ613568 | GII.12 | GII.P33 | HQ449728 | GII.12 | GII.P33 |
| KC464500 | GII.12 | GII.P33 | MK616561 | GII.12 | GII.P16 |
| MT712147 | GII.12 | GII.P33 | MK616560 | GII.12 | GII.P16 |
| MT712130 | GII.12 | GII.P33 | MK762627 | GII.12 | GII.P16 |
| KM198492 | GII.12 | GII.P33 | MK754445 | GII.12 | GII.P16 |
| KM198503 | GII.12 | GII.P33 | MK754447 | GII.12 | GII.P16 |
| KC464498 | GII.12 | GII.P33 | MK753036 | GII.12 | GII.P16 |
| KC464497 | GII.12 | GII.P33 | MK616559 | GII.12 | GII.P16 |
| KC464499 | GII.12 | GII.P33 | MK616558 | GII.12 | GII.P16 |
| JQ613569 | GII.12 | GII.P33 | MK355713 | GII.12 | GII.P16 |
| KC464496 | GII.12 | GII.P33 | MK355712 | GII.12 | GII.P16 |
| AB045603 | GII.P12 | GII.12 | KJ196299 | GII.P12 | GII.12 |
| AB039775 | GII.P12 | GII.12 | KJ196282 | GII.P12 | GII.12 |
| KJ196294 | GII.P12 | GII.12 | - | - | - |

Table S3. GenBank accession numbers and norovirus dual-typing information downloaded from Genbank for RdRp phylogenetic tree construction

| Genbank accession No. | RdRp | VP1 | Genbank accession No. | RdRp | VP1 |
| --- | --- | --- | --- | --- | --- |
| MK614135 | GII.P16 | GII.2 | NC_03947 | GII.P16 | GII.4 |
| MK614127 | GII.P16 | GII.2 | MK762566 | GII.P16 | GII.4 |
| MK614124 | GII.P16 | GII.2 | KY887601 | GII.P16 | GII.4 |
| MK614125 | GII.P16 | GII.2 | MK616561 | GII.P16 | GII.12 |
| MK614134 | GII.P16 | GII.2 | MK616560 | GII.P16 | GII.12 |
| MK614136 | GII.P16 | GII.2 | MK762627 | GII.P16 | GII.12 |
| MK614126 | GII.P16 | GII.2 | MK754445 | GII.P16 | GII.12 |
| MK614130 | GII.P16 | GII.2 | MK754447 | GII.P16 | GII.12 |
| MK614133 | GII.P16 | GII.2 | MK753036 | GII.P16 | GII.12 |
| MK614129 | GII.P16 | GII.2 | MK616559 | GII.P16 | GII.12 |
| MK614131 | GII.P16 | GII.2 | MK616558 | GII.P16 | GII.12 |
| MK614132 | GII.P16 | GII.2 | MK355713 | GII.P16 | GII.12 |
| MK614137 | GII.P16 | GII.2 | MK355712 | GII.P16 | GII.12 |
| MK614128 | GII.P16 | GII.2 | MK483908 | GII.P16 | GII.1 |
| MH041321 | GII.P16 | GII.2 | MG572182 | GII.P16 | GII.1 |
| MG745985 | GII.P16 | GII.2 | MK753033 | GII.P16 | GII.1 |
| MG892908 | GII.P16 | GII.13 | MK753034 | GII.P16 | GII.1 |
